# Automated sequential chromogenic IHC double staining with two HRP substrates

**DOI:** 10.1371/journal.pone.0207867

**Published:** 2018-11-20

**Authors:** Kenneth Heesche Petersen, Jesper Lohse, Lasse Ramsgaard

**Affiliations:** 1 Assay Teccology, Agilent Technologies, Glostrup, Denmark; 2 Product Design, Agilent Technologies, Glostrup, Denmark; Western College of Veterinary Medicine, University of Saskatchewan, CANADA

## Abstract

Automated IHC double staining using diaminobenzidine and HRP Magenta is illustrated utilizing a new acidic block with sulfuric acid to prevent cross-reactivity. Residual cross-reactivity in double staining is determined to arise from chromogenic-bound antibodies and amplification system during the first part of the double staining.

## Introduction

Obtaining enough information to make an informed diagnostic decision from a limited sample size is a major challenge with many diagnostic procedures today. For pathology labs, using immunohistochemistry (IHC), this is particularly important when the biopsy is small and only a few different tests can be run on the available tissue. The localization of different antigens in relation to each other can also be important for a diagnosis. In these cases, staining the same tissue section for two antigens is a useful diagnostic tool.

The possibility of cross-staining between two targets is commonly reduced by using antibodies derived from different species, usually rabbit and mouse [1]. In addition, visualization of the antigens is performed using different enzymes for the visualization steps. Typically, horseradish peroxidase (HRP) and alkaline phosphatase (AP) together with the substrates diaminobenzidine (DAB) and Fast Red, respectively, are used to obtain brown and red colors [2]. No quenching of enzymatic activity between the two stains is required as the two enzymes have different substrates. The colors brown and red contrast well to the commonly used blue hematoxylin nuclear stain.

However, there are drawbacks to the AP substrate system. Not only do Fast Red stains rapidly dissolve in ethanol and most organic mounting media, but the Fast Red diazonium salt can also react with a range of other substances present in the tissue section. This includes the DAB stain which becomes darker after incubation with Fast Red. Furthermore, Fast Red chromogen must be used within 30 minutes of mixing with the substrate. For automated staining systems, this requires onboard mixing of the Fast Red chromogen and substrate reagents. Alternatively, the staining is paused until the user can supply a freshly mixed Fast Red solution to the system.

An alternative chromogen, 3-amino-9-ethylcarbazole (AEC), exists for the HRP enzyme system, but this is not compatible with other peroxidase substrates due to poor color contrast between reddish-brown AEC and brown DAB [3].

Blue HRP substrates have previously been described. While they contrast well to DAB they are not optimal when hematoxylin is used as a counterstain [4].

Recently, a new magenta-colored chromogen for HRP with a sensitivity comparable to DAB has been described [5]. This paper describes a method for performing a fully automated DAB/Magenta chromogen IHC double stain using HRP to visualize both stains.

## Results and discussion

**Fig 1:**
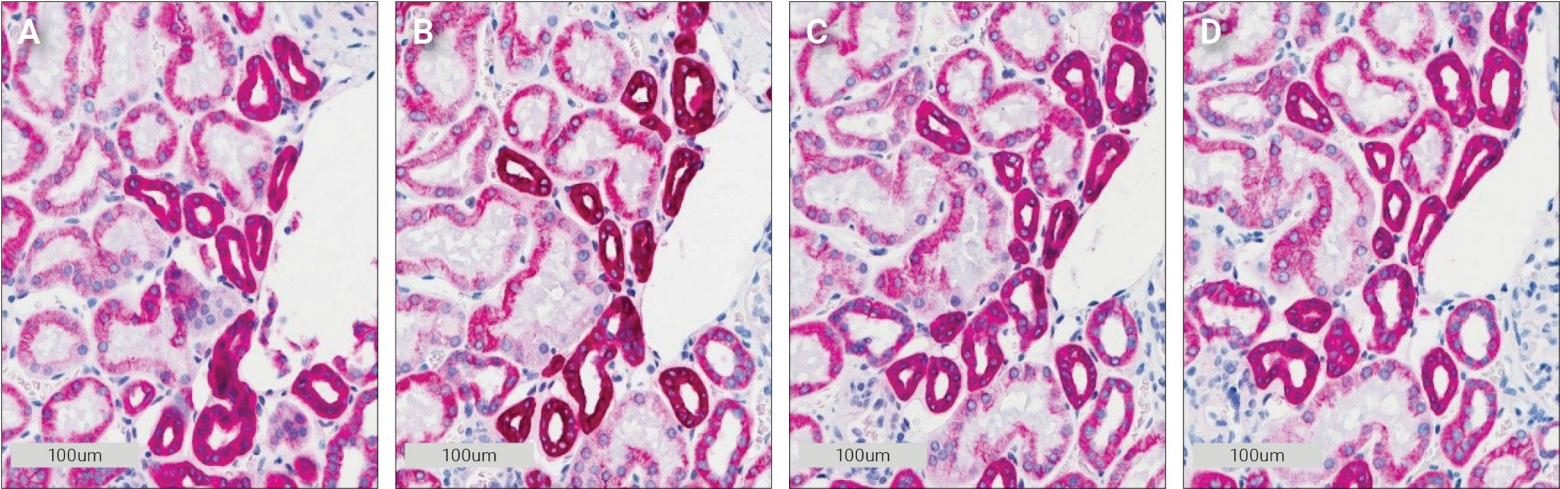
Effect of different blocking steps on cytokeratin Pan (CK Pan) staining in kidney tissue. **A** No block, no DAB. **B** Peroxidase block then DAB. **C** 50 mM H_2_SO_4_ then DAB. **D** 300 mM H_2_SO_4_ then DAB.

When performing double staining, it is important to eliminate any cross-reactivity between reagents. In the present study, two chromogens that are both substrates for the HRP enzyme are used. This makes it important to efficiently remove all enzyme activity from the first stain before performing the second stain. Peroxidase blocking is normally performed to quench any endogenous peroxidase activity in the tissue [6]. This does not, however, remove all HRP activity when the tissue contains additional peroxidase activity from the visualization conjugates as seen in Fig 1B, so additional removal of peroxidase activity is necessary. Sequential double staining using two HRP enzymes has previously been described using an acidic block between the two stains [7]. An acidic block works by dissociating the antibodies from their target antigens, so they can be washed off.

### Sulfuric acid treatment

The efficiency of using an acidic block was tested using sulfuric acid. Starting from 300 mM sulfuric acid, H_2_SO_4,_ a two-fold dilution series down to 20 mM sulfuric acid was made and tested with four different antibodies. The acid block step was set to 3 minutes, which is the minimum time on the automated Dako Omnis staining system for a block or incubation step.

The test was set up as a single antibody stain, with two consecutive chromogenic stains. First, HRP Magenta chromogen followed by a 3-minute acid block, wash buffer and then a second DAB chromogen incubation. No DAB staining was visible on any of the slides, thus the acidic block removed all detectable enzyme activity and the morphology of the tissue was not visibly impacted (Fig 1C and 1D).

**Fig 2:**
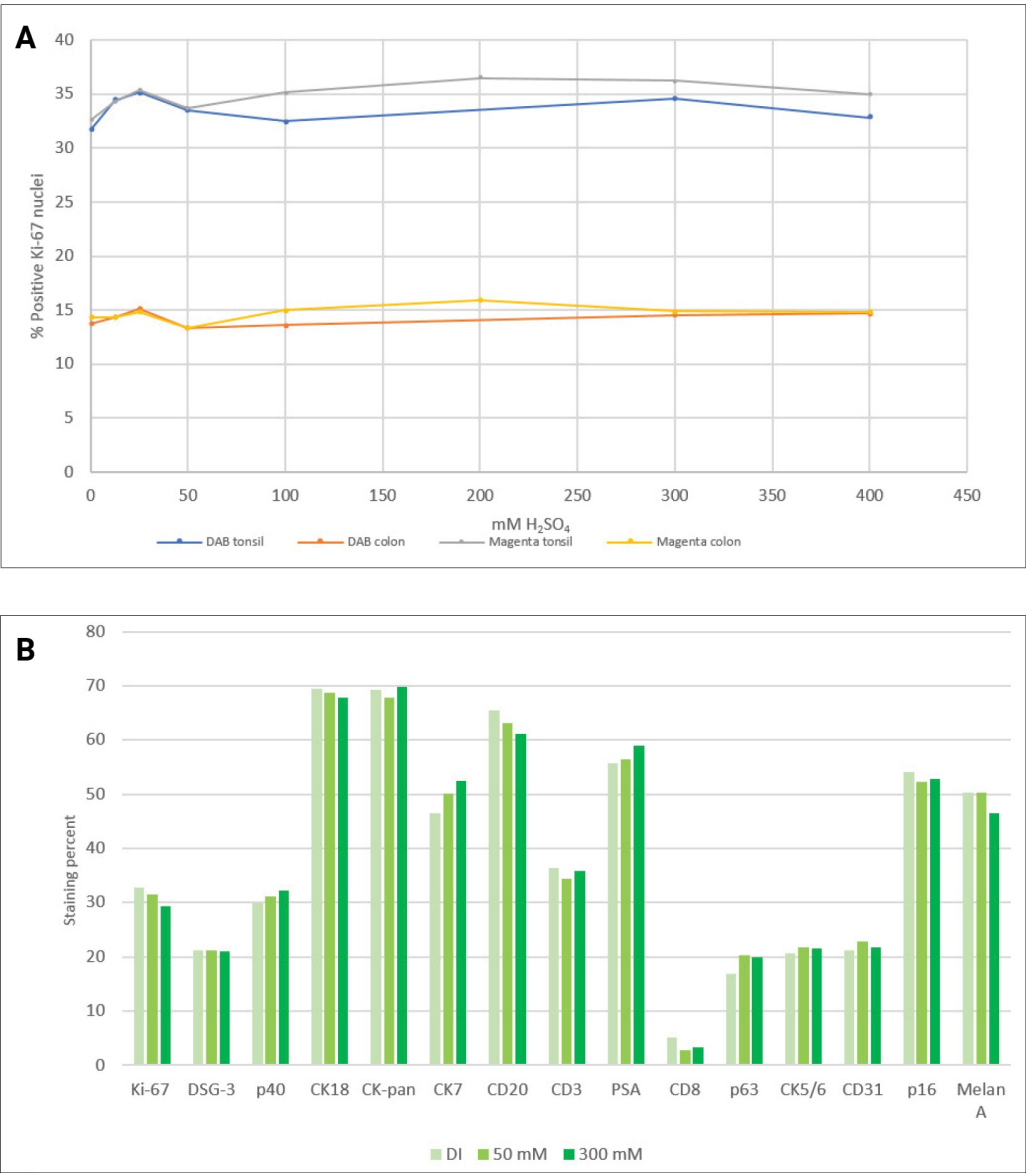
Effect of sulfuric acid on staining intensity. **A** Percent Ki-67 positive nuclei in tonsil and colon using either DAB or Magenta as chromogen. **B** Effect of sulfuric acid block before IHC staining and with Magenta as chromogen reported as either percent stained nuclei or percent stained complete membranes (DI = deionized water). More than 10.000 cells were analyzed for every single data point.

Next, it was investigated if either of the two chromogens from a completed staining was affected by a sulfuric acid block. Ki-67 stains of nuclei in tonsil and colon tissue were used for this experiment. Ki-67-stained nuclei can be counted using an image analysis algorithm. Slides stained with either DAB or HRP Magenta were subjected to a subsequent block with sulfuric acid, in concentrations ranging from 0 to 400 mM. The percentage of Ki-67 positive nuclei in tonsil and colon was counted for each concentration of sulfuric acid. Fig 2 shows that the tested concentrations have no effect on the staining of the slides as the chromogen already present on the slides is not removed or faded by the acid block. It is concluded that sulfuric acid in the range 50–400 mM effectively removes residual HRP activity, without negatively impacting the intensity of a first DAB or HRP Magenta stain.

Some epitopes may be sensitive to the sulfuric acid block which could in turn affect the intensity of the second staining. This was investigated by applying an acid step prior to application of the primary antibody and completing the staining as a single stain with either DAB or HRP Magenta chromogen. Three different treatments before primary antibody were compared: Block with deionized water, 50 mM- or 300 mM sulfuric acid. Sixteen different antibodies were used, and stains were evaluated using digital image analysis (Fig 2B). Manual scoring of the analyzed slides confirmed that only minor changes in staining intensity could be seen. These minor changes were within the normal variation we commonly observe when manually scoring IHC-stained slides.

The stains in the current study were used qualitatively and the effect (if any) is therefore negligible. It is, however, important to be aware of any impact on staining intensity from either the first staining or the acid block if a double staining is to be used for quantification.

Moving forward with the double staining, a belt-and-braces approach was used with sulfuric acid first, followed by a peroxidase block to ensure that all remaining peroxidase activity was quenched. The two quenching reagents are orthogonal; the acidic block dissociates the antibodies from their targets while the peroxidase block works by ‘overloading’ the enzymes with hydrogen peroxide they cannot get rid of as no substrate is present. This eventually quenches the enzyme activity. Sulfuric acid was placed first in the second part of the staining protocol to remove as much bound antibody as possible before remaining antibody-enzyme conjugate still in the tissue were quenched by treatment with peroxidase block.

### Non-co-localized targets

**Fig 3:**
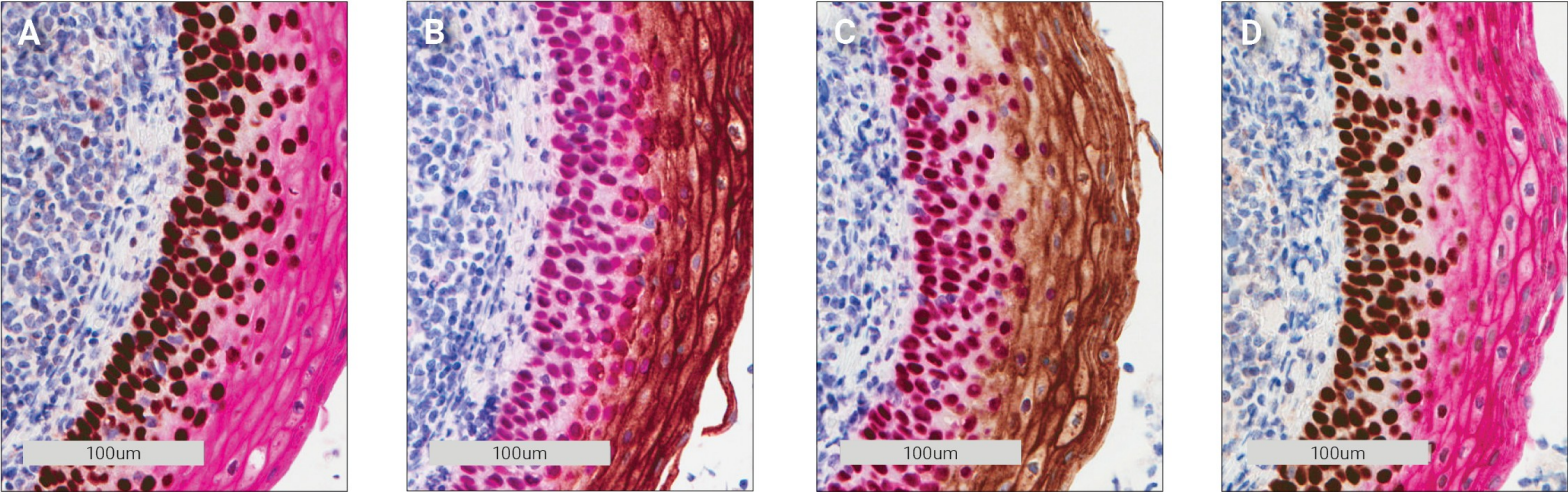
Carcinoembryonic antigen (CEA) and p63 double staining of tonsil tissue using monoclonal mouse antibodies. **A** 1st. p63/DAB 2nd. CEA/Magenta. **B** 1st. CEA/DAB 2nd. p63/Magenta. **C** 1st. p63/Magenta 2nd. CEA/DAB. **D** 1st. CEA/Magenta 2nd. p63/DAB.

A double staining was performed with monoclonal mouse antibodies against a nuclear target (p63) and a membrane target (carcinoembryonic antigen (CEA)), respectively. The stains were expected to be unaffected by each other since the two antigens are not co-localized. Stainings using the same combination of antibodies were run with HRP Magenta first then DAB and vice versa. Fig 3A–3D show these double stains.

Cross-reactivity can be seen when DAB is used as the first chromogen, either as a reddish ring around the nuclei (Fig 3A) or as a change in the brown color (Fig 3B). Likewise, when the magenta color is used first, it becomes a darker red (Fig 3C and 3D). The purple color of the nuclei in Fig 3B is expected since it is a combination of the magenta and the blue hematoxylin colors.

**Fig 4:**
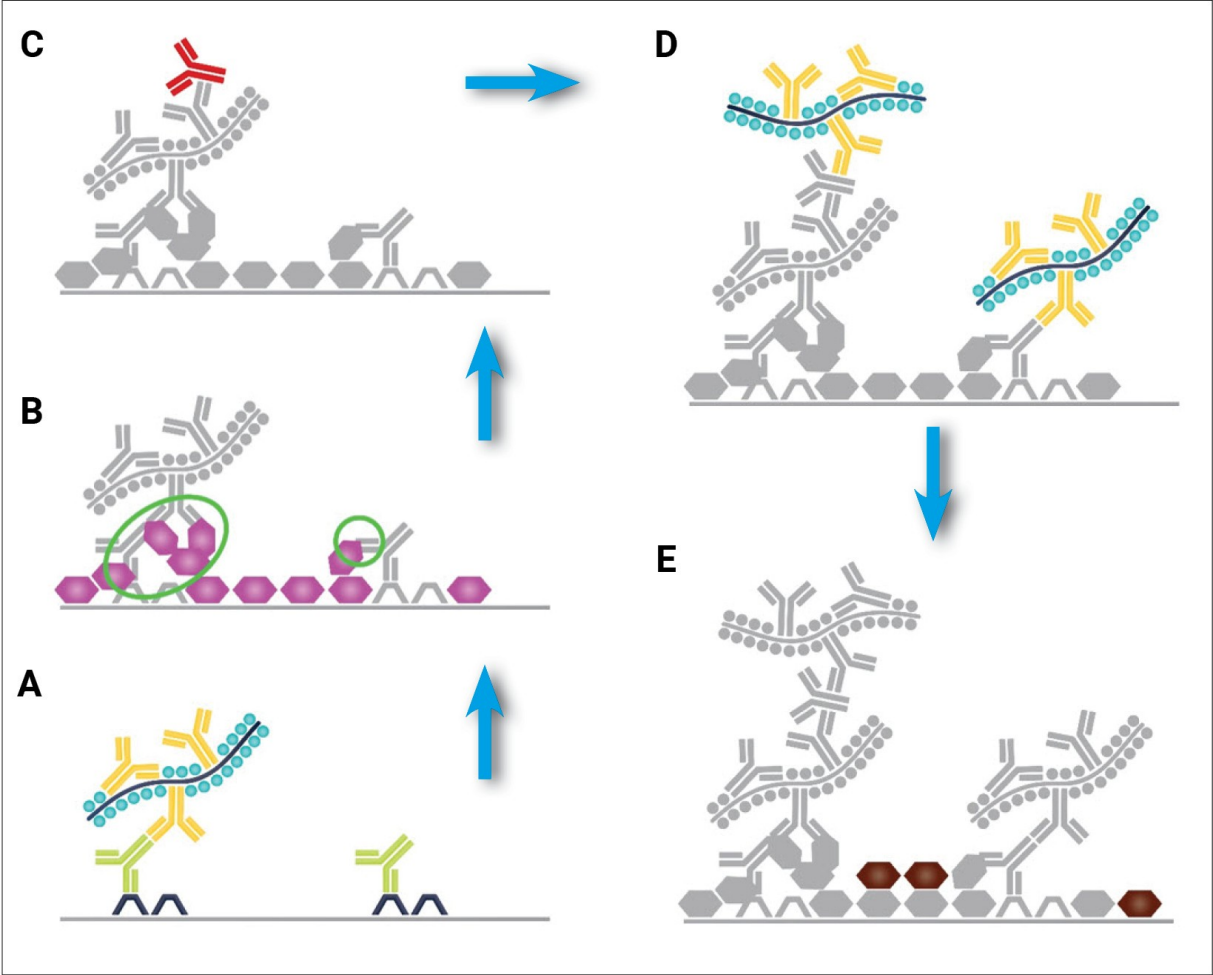
Cross-reactivity through irreversible binding of antibodies. **A** The first primary antibody binds to its antigen and is recognized by the amplification system. **B** Chromogen is precipitated and covalently binds antibodies and amplification system to the tissue, green circles. **C** The second primary antibody is captured by free antibodies of the amplification system. **D** As the second layer of amplification is applied, antibodies captured by the first amplification layer, along with those covalently bound to the tissue are captured. **E** color spillover results when the next chromogen is applied.

The observed color spillover was not expected because the first experiments demonstrated that all peroxidase activity in the tissue had been removed following sulfuric acid and peroxidase block. One possible explanation for this unexpected color spillover is that reagents from the first stain remain in the tissue because the chromogen has covalently bonded them to the tissue (Fig 4A–4E). When the second stain is performed the reagents are recognized by remaining amplification system (Fig 4C) and remaining primary antibodies are recognized by the amplification system along with the primary antibodies captured by the remaining amplification system (Fig 4D). The chromogen is then precipitated during the second chromogen incubation (Fig 4E) which causes the color spillover.

**Fig 5:**
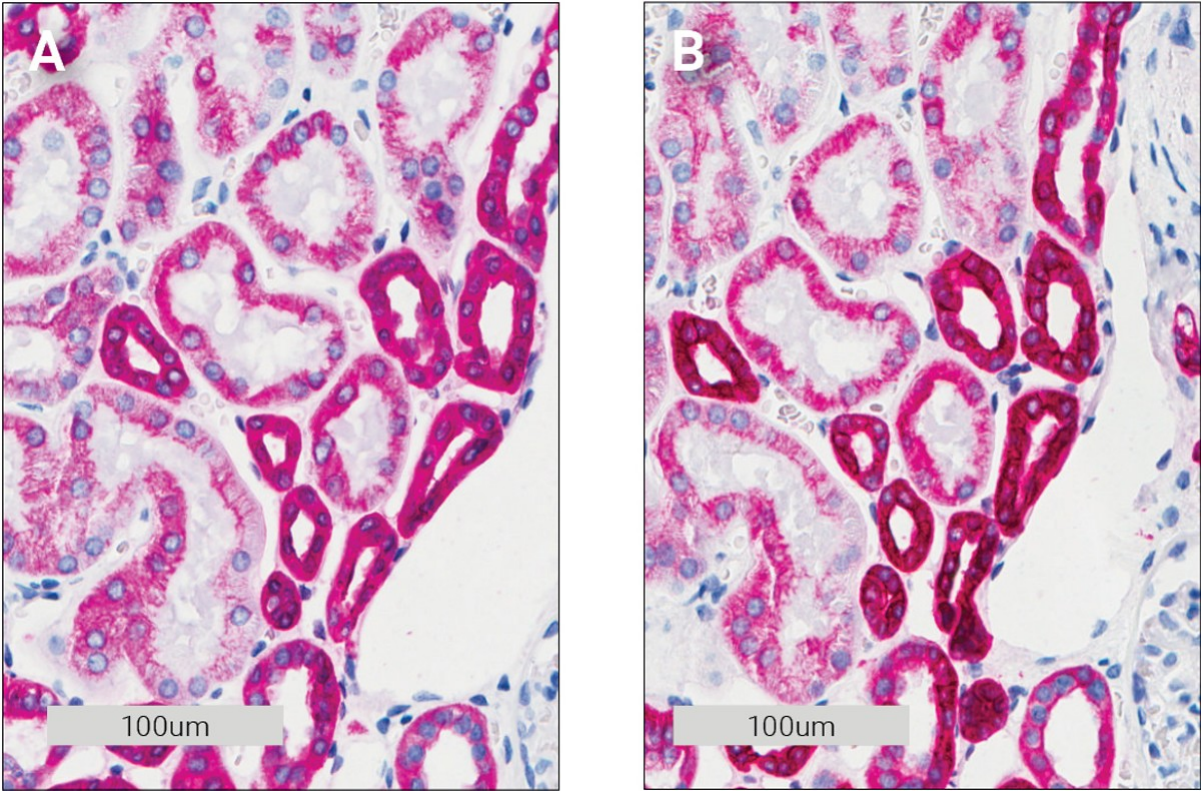
Demonstration of residual bound antibodies in kidney tissue after sulfuric acid and peroxidase block treatment. **A** Reference 300 mM sulfuric acid block then DAB (same picture as Fig 1D). **B** 300 mM sulfuric acid, peroxidase block then amplification.

To test our theory, the initial sulfuric acid and peroxidase block experiments were expanded. A complete stain for CK Pan with HRP Magenta was performed first and then the slide was treated with 300 mM sulfuric acid and peroxidase block to remove all remaining peroxidase activity. This was followed by an incubation with the amplification system once more before a final DAB incubation. As seen by comparing Fig 5A with 5B, the magenta color became visibly tainted by DAB (it is darker). While this does not rule out other explanations it does support the theory that reagents from the first stain remain in the tissue.

**Fig 6:**
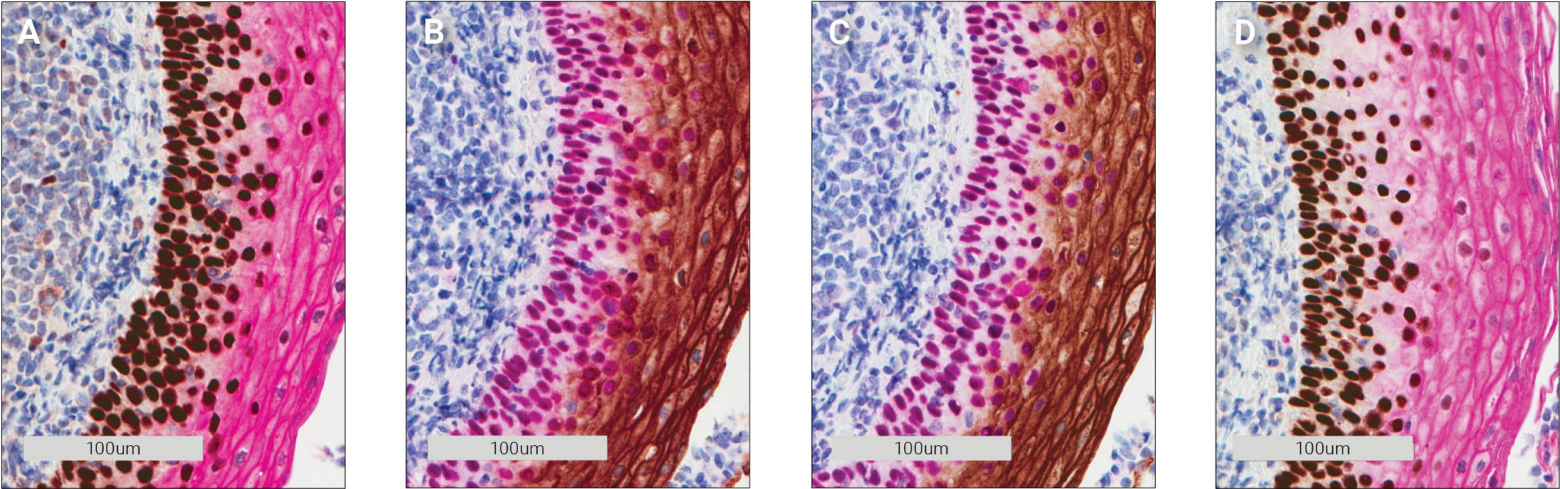
Rabbit polyclonal CEA and mouse monoclonal p63 antibody double staining of tonsil tissue. **A** 1st. p63/DAB 2nd. CEA/Magenta. **B** 1st. CEA/DAB 2nd. p63/Magenta. **C** 1st. p63/Magenta 2nd. CEA/DAB. **D** 1st. CEA/Magenta 2nd. p63 DAB.

The double staining of p63 and CEA was repeated, this time using a rabbit antibody against CEA while the p63 antibody was the same mouse antibody as in the initial experiment. As seen in Fig 6A–6D the colors of the double stainings became untainted, regardless of which chromogen was used as the first chromogen (compare Fig 3B with Fig 6B and Fig 3D with Fig 6D).

### Co-localized targets

**Fig 7:**
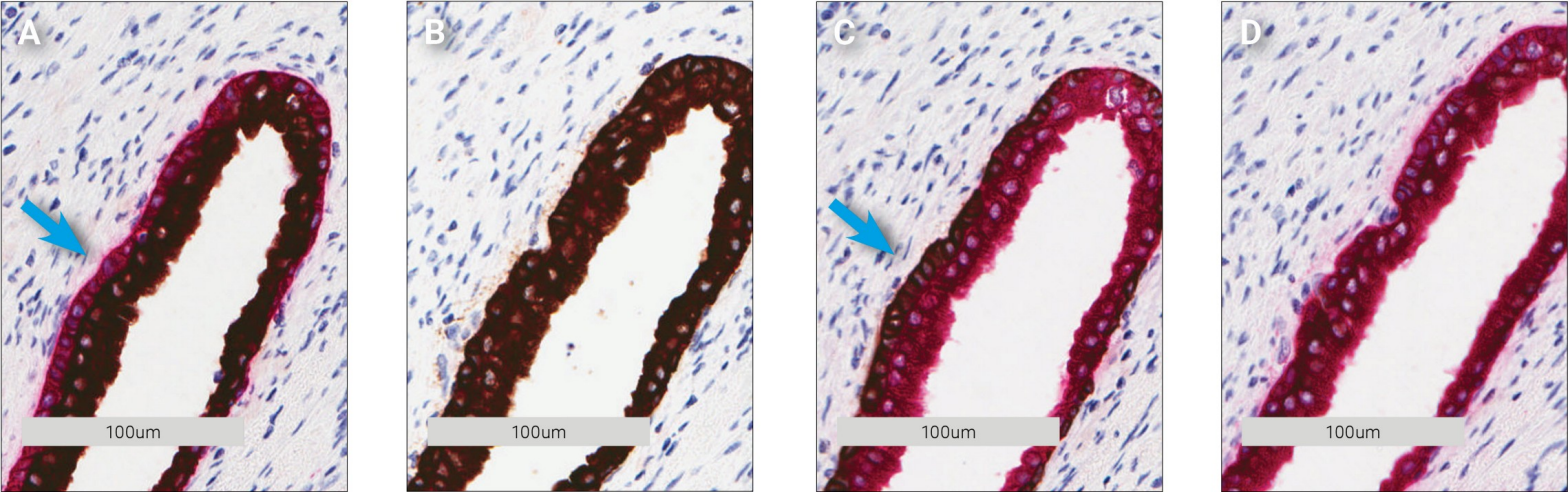
CK Pan/CK 18 double staining of prostate tissue on Dako Omnis. **A** 1st. CK 18/DAB 2nd. CK Pan/Magenta. **B** 1st. CK Pan/DAB 2nd. CK 18/Magenta. **C** 1st. CK 18/Magenta 2nd. CK Pan/DAB. **D** 1st. CK Pan/Magenta 2nd. CK 18/DAB. The corresponding single antibody stains are in the supplementary material.

The next step was to investigate double staining protocols with co-localized targets. For this experiment, CK Pan and cytokeratin 18 (CK 18) antibodies were used. CK 18 is not recognized by the CK Pan antibody but is co-localized with CK Pan in prostate glands. Fig 7A–7D shows the result from the cytokeratin double stainings. The CK Pan antibody stains a slightly larger area of the prostate epithelium than CK 18. This resulted in a rim of either HRP Magenta-stained CK Pan (Fig 7A) or DAB-stained CK Pan around the CK 18 stain (Fig 7C). The rim was only visible when CK 18 is used as the first antibody in the double staining protocol. If the larger CK Pan area was stained first, a shielding of the co-localized CK 18 target came into effect. This markedly lowered the staining intensity of CK 18. In the case where DAB was used together with CK Pan, the co-localization of the second target was not easily seen (Fig 7B). The transparency of the magenta color makes it comparably easier to see that the targets are co-localized. Although the HRP Magenta/DAB combination is not ideal for co-localized targets, using the more transparent HRP Magenta as the first chromogen increases the likelihood that co-localized targets are detected due to tainting of the magenta color. When DAB was used as the first chromogen only the expected areas for each antibody were stained and the colors were not visibly tainted. The observed difference is attributed to the epitope shielding effect. The DAB precipitate can be so dense that access to epitopes in the tissue by subsequent antibodies is almost impossible. This, combined with the ability of the brown color to dominate other colors, keeps the DAB stain brown (Fig 7A) even after the second staining is complete. The same is true in Fig 7B where the DAB stain kept its brown appearance but was lighter due to some HRP Magenta precipitation. This is ascribed to the different expression levels of cytokeratin, which affects how dense the DAB precipitate becomes.

The situation is the same for the HRP Magenta chromogen, with a high shielding effect observed when CK 18 was stained first and less shielding when CK Pan was stained first.

## Conclusion

We have demonstrated double staining using two HRP substrates as chromogens. The HRP activity from the first staining can be completely removed by a sulfuric acid block step. Using the HRP Magenta chromogen in combination with DAB makes fully automated protocols for double staining possible.

The best double staining in terms of no cross-reactivity is obtained with antibodies from two different species. However, in cases of densely packed epitopes and when using DAB as the first chromogen, the spillover is practically hidden by the intense DAB stain. This makes it possible to do a double staining with two antibodies from the same species. When using antibodies from different species, the sequence of the chromogens is not important, and both DAB- and HRP Magenta chromogen can be used as the first stain. We have explained that cross-reactivity remains an issue when using antibodies from the same species. We propose that this crossreactivity is due to covalently bound antibodies from the first stain.

In our experience the use of DAB as the first chromogen and HRP Magenta as the second chromogen have given consistently satisfactory results with minimal detectable cross reactivity and this is now our standard approach when setting up double staining protocols.

## Materials and methods

Chemicals were purchased from Sigma Aldrich. Antibodies were purchased from Agilent Technologies, Inc (Glostrup, Denmark), except Desmoglein-3, p40 and p16 antibodies which were from Abcam Inc. (Cambridge,MA, USA), Biocare Medical, Inc. (Pacheco, CA, USA) and Santa Cruz Biotechnology, Inc. (Dallas Texas, USA), respectively. All antibodies were used according to the manufacturer’s specifications. Automated staining was performed on either a Dako Omnis system using the “IHC Double Stain Template” or an Autostainer Link 48 system (See supporting information). On the Dako Omnis system the “IHC Double Stain Template” was used. For the Autostainer Link 48, a protocol was defined. The protocols can be found in the supporting information along with the incubation times for the antibodies used.

Images were captured on an Aperio Scanscope. Areas of interest were loosely circled and analyzed using the membrane v9 or the nuclear v9 algorithm adjusted to the color of the chromogen.

### Specimens

All tissue was obtained from Department of Pathology, Odense University Hospital, Region South, Denmark, fixed in formaldehyde for ~24 hours prior to embedding in paraffin. All specimens were completely anonymized prior to receipt at Agilent. According to the Danish law on the Research Ethics Committee System and handling of biomedical research projects and communication between Agilent and the Danish Committee on Biomedical Research Ethics and the Regional Ethics Committee (IRB), the tests performed at Agilent on anonymous residual tissue are not subject to an approval by the IRB system because such studies are considered quality control projects. Therefore, no IRB approval for this work has been obtained.

## Supporting information

S1 Table Autostainer link 48 protocol. (DOCX)

S2 Table Dako Omnis protocol. (DOCX)

S3 Table Antibody protocol information. (DOCX)

S1 Fig Cytokeratin Pan/18 single stains. (DOCX)
