# Supplementary material for: Automated sequential chromogenic IHC double staining with two HRP substrates
Source: PLoS One. 2018 Nov 20;13(11):e0207867. doi: 10.1371/journal.pone.0207867 (PMC6245840; doi:10.1371/journal.pone.0207867)
Supplement: S1 Table — (DOCX) [file pone.0207867.s001.docx]

Supporting information

**S1 Table.** **Autostainer Link 48 protocol**

| **Step** | **Category** | **Reagent** | **Incubation** |
| --- | --- | --- | --- |
| 1 | Rinse | Buffer | 0 min |
| 2 | Endogenous Enzyme Block | FLEX Peroxidase Block | 5 min |
| 3 | Rinse | Buffer | 0 min |
| 4 | Primary Antibody | 1. primary antibody | Individual |
| 5 | Rinse | Buffer | 0 min |
| 6 | Secondary Reagent* | FLEX+ (LINKER) | 15 min |
| 7 | Rinse* | Buffer | 0 min |
| 8 | Labelled Polymer | FLEX /HRP | 20 min |
| 9 | Rinse | Buffer | 0 min |
| 10 | Rinse | Buffer | 5 min |
| 11 | Substrate-Chromogen | FLEX DAB+ Sub-Chromo | 5 min |
| 12 | Substrate-Chromogen | FLEX DAB+ Sub-Chromo | 5 min |
| 13 | Rinse | Buffer | 0 min |
| 14 | Endogenous Enzyme Block | H_2_SO_4_, 50-300 mM | 3 min |
| 15 | Rinse | Buffer | 0 min |
| 16 | Endogenous Enzyme Block | FLEX Peroxidase Block | 5 min |
| 17 | Rinse | Buffer | 0 min |
| 18 | Primary Antibody | 2. primary antibody | Individual |
| 19 | Rinse | Buffer | 0 min |
| 20 | Secondary Reagent* | FLEX+ (LINKER) | 15 min |
| 21 | Rinse* | Buffer | 0 min |
| 22 | Labelled Polymer | FLEX /HRP | 20 min |
| 23 | Rinse | Buffer | 0 min |
| 24 | Rinse | Buffer | 5 min |
| 25 | Substrate-Chromogen | HRP-Magenta | 5 min |
| 26 | Rinse | Buffer | 0 min |
| 27 | Counterstain | FLEX Hematoxylin | 5 min |
| 28 | Rinse | DI Water | 0 min |
| 29 | Rinse | Buffer | 5 min |
| 30 | Rinse | DI Water | 0 min |

* Only applies if LINKER is use.
