# Supplementary material for: Automated sequential chromogenic IHC double staining with two HRP substrates
Source: PLoS One. 2018 Nov 20;13(11):e0207867. doi: 10.1371/journal.pone.0207867 (PMC6245840; doi:10.1371/journal.pone.0207867)
Supplement: S2 Table — (DOCX) [file pone.0207867.s002.docx]

Supporting information

**S2 Table. Dako Omnis protocol**

| **Step** | **Category** | **Reagent** | **Incubation** |
| --- | --- | --- | --- |
| 1 | Wash | Wash buffer | 2:40 min |
| 2 | Primary antibody | 1. primary antibody | Individual |
| 3 | Wash | Wash buffer | 2 min |
| 4 | Endogenous enzyme block | EnV FLEX Peroxidase-Blocking Reagent | 3 min |
| 5 | Wash | Wash buffer | 2 min |
| 6 | Secondary reagent* | EnV FLEX+ LINKER | 10 min |
| 7 | Wash* | Wash buffer | 2 min |
| 8 | Labeled polymer | EnV FLEX/HRP | 20 min |
| 9 | Wash | Wash buffer | 2 min |
| 10 | Wash | Wash buffer | 2 min |
| 11 | Wash | DI water | 31 sec |
| 12 | Wash | Wash buffer | 2 min |
| 13 | Substrate chromogen | EnV FLEX Substrate  Working Solution | 5 min |
| 14 | Wash | Wash buffer | 2 min |
| 15 | Wash DI water | DI water | 31 sec |
| 16 | Wash | Wash buffer | 2 min |
| 17 | Generic endogenous enzyme block | H_2_SO_4_, 50-300 mM | 3 min |
| 18 | Wash | Wash buffer | 2 min |
| 19 | Primary antibody | 2. primary antibody | Individual |
| 20 | Wash | Wash buffer | 2 min |
| 21 | Endogenous enzyme block | EnV FLEX Peroxidase-Blocking Reagent | 3 min |
| 22 | Wash | Wash buffer | 2 min |
| 23 | Secondary reagent* | EnV FLEX+ LINKER | 10 min |
| 24 | Wash* | Wash buffer | 2 min |
| 25 | Labeled polymer | EnV FLEX/HRP | 20 min |
| 26 | Wash | Wash buffer | 2 min |
| 27 | Wash | Wash buffer | 2 min |
| 28 | Wash | DI water | 31 sec |
| 29 | Wash | Wash buffer | 2 min |
| 30 | Substrate chromogen | EnV FLEX HRP Magenta  Substrate Working Solution | 5 min |
| 31 | Wash | Wash buffer | 2 min |
| 32 | Wash | DI water | 31 sec |
| 33 | Wash | Wash buffer | 2 min |
| 34 | Counterstain | Hematoxylin | 3 min |
| 35 | Wash | DI water | 2 min |
| 36 | Wash | Wash buffer | 2 min |

* Only applies if LINKER is used
