# Supplementary material for: Automated sequential chromogenic IHC double staining with two HRP substrates
Source: PLoS One. 2018 Nov 20;13(11):e0207867. doi: 10.1371/journal.pone.0207867 (PMC6245840; doi:10.1371/journal.pone.0207867)
Supplement: S3 Table — (DOCX) [file pone.0207867.s003.docx]

Supporting information

**S3 Table Antibody protocol information**

| **Product** | **Clone** | **Source** | **Dilution** | **Diluent** | **Detection System** | **Target Retrieval** **pH** | **Primary** **Antibody** | **Linker** **Antibody** | **EnVision** **FLEX /HRP** |
| --- | --- | --- | --- | --- | --- | --- | --- | --- | --- |
| CD3 | Polyclonal | Agilent Technologies | RTU | - | FLEX+ Rabbit | Low pH | 20 min | 10 min | 20 min |
| CD8 | C8/114B | Agilent Technologies | RTU | - | FLEX+ Mouse | High pH | 10 min | 10 min | 20 min |
| CD20cy | L26 | Agilent Technologies | RTU | - | FLEX+ Mouse | High pH | 12.5 min | 10 min | 20 min |
| CD31 | JC70A | Agilent Technologies | RTU | - | FLEX+ Mouse | High pH | 20 min | 10 min | 20 min |
| CK 5/6 | D5/16 B4 | Agilent Technologies | RTU | - | FLEX | High pH | 12.5 min | None | 20 min |
| CK 7 | OV-TL 12/30 | Agilent Technologies | RTU | - | FLEX | High pH | 12.5 min | None | 20 min |
| CK 18 | DC 10 | Agilent Technologies | RTU | - | FLEX | High pH | 25 min | None | 20 min |
| CK-Pan | AE1/AE3 | Agilent Technologies | RTU | - | FLEX | High pH | 12.5 min | None | 20 min |
| CEA | II-7 | Agilent Technologies | RTU | - | FLEX+ Mouse | High pH | 25 min | 10 min | 20 min |
| Desmoglein-3 | 5G11 | Abcam | 1:15 | S0809 | FLEX+ Mouse | Low pH | 20 min | 10 min | 20 min |
| Ki-67 | MIB-1 | Agilent Technologies | RTU | - | FLEX | High pH | 20 min | None | 20 min |
| Melan A | A103 | Agilent Technologies | 5.5 µg/mL | S0809 | FLEX+ Mouse | High pH | 20 min | 10 min | 20 min |
| p16 | JC8 | Santa Cruz Biotechnology | 0.5 µg/mL | S0809 | FLEX+ Mouse | High pH | 20 min | 10 min | 20 min |
| p40 | BC28 | Biocare | 1:25 | S0809 | FLEX+ Mouse | Low pH | 20 min | 10 min | 20 min |
| p63 | DAK-p63 | Agilent Technologies | 1 µg/mL | S0809 | FLEX | High pH | 20 min | None | 20 min |
| PSA | Polyclonal | Agilent Technologies | RTU | - | FLEX | High pH | 12.5 min | None | 20 min |
