# Supplementary material for: Automated sequential chromogenic IHC double staining with two HRP substrates
Source: PLoS One. 2018 Nov 20;13(11):e0207867. doi: 10.1371/journal.pone.0207867 (PMC6245840; doi:10.1371/journal.pone.0207867)
Supplement: S1 Fig — (DOCX) [file pone.0207867.s004.docx]

**Supporting information**


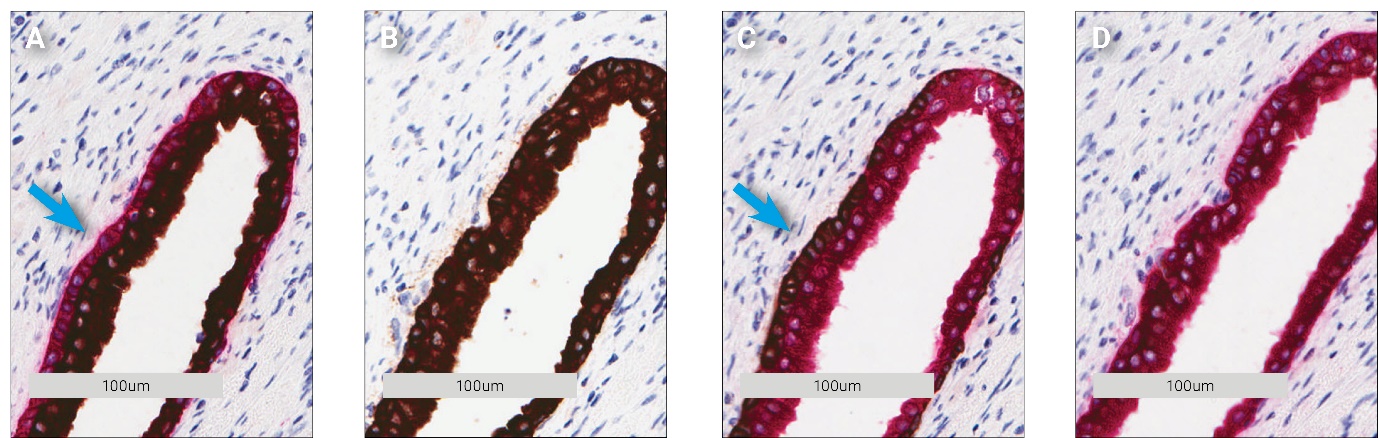


**Figure S1.** Single antibody stains **A** CK-18/DAB **B** CK-Pan/Magenta **C** CK-Pan/DAB **D** CK-18/Magenta
